# The “double-edged” role of progesterone in periodontitis among perimenopausal women undergoing or not undergoing scaling and root planing

**DOI:** 10.3389/fendo.2023.1224763

**Published:** 2023-08-14

**Authors:** Shengjie Yan, Ying Man, Jun Lu, Liyun Cui, Feifei Niu, Jianyong Qin

**Affiliations:** ^1^ Department of Stomatology, Shengli Oilfield Central Hospital, Dongying, China; ^2^ Department of Gynaecology, Shengli Oilfield Central Hospital, Dongying, China; ^3^ Department of Stomatology, The Affiliated Hospital of Qingdao University, Qingdao, China

**Keywords:** progesterone, perimenopause, periodontitis, inflammation, scaling and root planning

## Abstract

**Objective:**

Progesterone (PG) is an important sex steroid hormone commonly administered to protect the endometrium in perimenopausal women. The present study aimed to explore differential responses of periodontitis to PG in perimenopausal women who did or did not undergo scaling and root planing (SRP).

**Methods:**

A total of 129 perimenopausal women with mild-to-moderate periodontitis were enrolled and underwent treatment as follows: SRP (n = 35); SRP + PG (n = 34); PG (n = 31); and no treatment (s) (n = 29). Pocket probing depth (PPD), clinical attachment level (CAL), sulcus bleeding index (SBI), and bleeding on probing (BOP) were measured using periodontal probes. Three inflammatory markers, including C-reactive protein (CRP), interleukin (IL)-6, and tumor necrosis factor-alpha (TNF-α) in gingival crevicular fluid (GCF) were measured using ELISA techniques.

**Results:**

PPD, CAL, SBI, BOP, and levels of inflammatory factors in GCF were all significantly decreased in perimenopausal women with periodontitis after SRP. In patients who did not undergo SRP, 6 months of PG treatment significantly elevated PPD, SBI, BOP, and GCF levels of CRP, IL-6, and TNF-α. In contrast, PG exhibited inhibitory effects on periodontal inflammation in patients who underwent SRP, evidenced by significantly decreased BOP and IL-6, and slightly decreased SBI, CRP, and TNF-α. PG-induced changes dissipated 6 months after withdrawal of PG (at 12 months).

**Conclusions:**

Among perimenopausal women with periodontitis, PG enhanced periodontal inflammation in the absence of SRP but inhibited periodontal inflammation in those who underwent SRP.

## Introductions

Periodontitis is a common inflammatory disease characterized by progressive destruction of the periodontal ligament and alveolar bone (1). Globally, the total prevalence of periodontitis has increased by 99.0% (2), and the age-standardized prevalence of severe periodontitis increased by 8.44% from 1990 to 2019 (3). The etiology of periodontitis is multifactorial and various risk factors have been identified, including old age, smoking, and diabetes. Mental disorders, drug abuse, poor dental care, and unhealthy dietary and lifestyle habits are also associated with periodontitis (4). Dynamic interactions between specific bacterial pathogens and the host inflammatory and immune responses are involved in the pathogenesis of periodontitis (5). Clinically, the principle of treating periodontitis is primarily to block the inflammatory process by controlling infection (6). Home care, and scaling and root planing (SRP) are the primary nonsurgical strategies, and regular long-term supportive therapy is crucial for good outcomes (7). If not treated appropriately and/or, in a timely manner, periodontitis may ultimately lead to tooth loss, thus greatly reducing quality of life (7, 8).

Because the periodontium is a target tissue for sex hormones, hormone-related events in females, such as pregnancy and menopause, are also important factors involved in the occurrence and progression of periodontitis (9–11). Perimenopause represents a transitional period of fluctuation(s) in sex hormone levels, indicating the initiation of reproductive senescence (12). Obvious physiological, emotional, and psychological changes occur in women during the perimenopausal period, along with a series of other problematic symptoms (13, 14). Notably, periodontal diseases, including periodontitis, tend to occur during the perimenopausal period because changes in sex hormone levels significantly influence the inflammatory response, bone resorption, and bone mineral density (15, 16). Gil-Montoya et al. reported that moderate or severe periodontitis presents in 52.6% of perimenopausal women, and that low bone mineral density in females > 58 years of age is positively associated with the onset of periodontitis (10). Chandra et al. reported that the perimenopausal-postmenopausal transition leads to an increasing oxidative stress in periodontal tissues and gingival crevicular fluid (GCF) (17). Manual intervention during perimenopause, therefore, is necessary to reduce the risk for periodontitis.

Estrogen and progesterone (PG) are the two most important sex hormones in females. During the normal menstrual cycle, PG physiologically antagonizes the effects of estrogen (18, 19). However, the production of PG is limited due to the absence of corpus luteum in the perimenopausal period (20). Without sufficient PG, estrogen may stimulate endometrial proliferation, leading to endometrial hyperplasia and carcinoma (21). Thus, PG is commonly administered to protect the endometrium in perimenopausal women. Interestingly, evidence has supported multiple roles for PG in the periodontal environment. PG can promote bone formation, exhibiting potential for the prevention and treatment of menopause-related osteoporosis (22). However, PG enhances chronic reactions by acting as an immunosuppressant in gingival tissues, resulting in exaggerated inflammation (23–25). Nevertheless, the specific role of PG in periodontitis among perimenopausal women has rarely been reported.

In the present study, perimenopausal women with periodontitis who were treated with SRP and/or PG (6 months) were enrolled. Differential responses of periodontitis to PG were evaluated in patients who did or did not undergone SRP. The study aimed to reveal the differential effects of routine PG intervention on the progression of periodontitis among perimenopausal women, in the presence or absence of SRP.

## Methods

### Subjects

**Figure 1:**
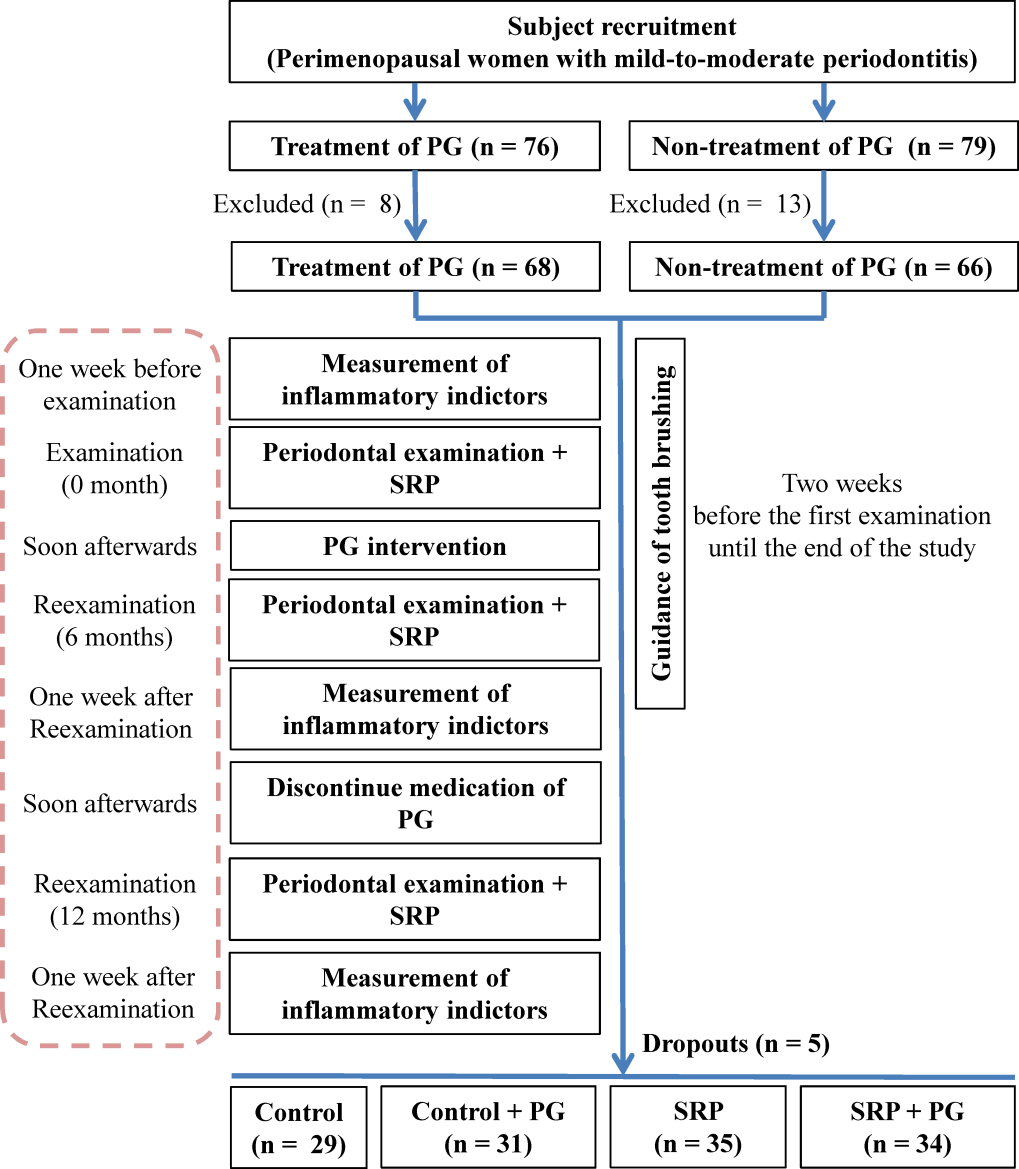
A flowchart of the study design.

This was a prospective cohort study. A total of 76 perimenopausal women (45–55 years of age) with mild-to-moderate chronic periodontitis and requiring PG were screened from the Department of Gynecology, Shengli Oilfield Central Hospital (Dongying, Shandong, China) between January 2021 and January 2022. Correspondingly, 79 perimenopausal women with mild-to-moderate chronic periodontitis who did not receive PG were recruited from the Physical Examination Center of the authors’ hospital in the same period. Periodontitis was diagnosed according to the Classification of Periodontal and Peri-Implant Diseases and Conditions described in 2017 (26). Inclusion criteria were as follows: fulfill the diagnostic criteria for mild-to-moderate chronic periodontitis; ≥ 20 teeth in the mouth; and 45–55 years of age at the perimenopausal period. Individuals with a history of periodontal therapy within 1 year before enrollment, medication history of antibiotics and/or immunosuppressants within 6 months, systemic and/or infectious diseases, and/or smoking history were excluded from this study. During the one-year study period, 5 patients were lost to follow-up. Ultimately, 129 patients were grouped as follows: SRP combined with 6 months of PG (SRP + PG [n = 34]); 6 months of PG (Control + PG [n = 31]); SRP (n = 35); and no treatment (Control [n = 29]). A flowchart illustrating the study design is presented in **Figure 1**. The present study was approved by the Ethics Committee of Shengli Oilfield Central Hospital in accordance with the Declaration of Helsinki. Written informed consent was obtained from all patients (Q/ZXYY-ZY-YWB-LL201819).

### Treatments

For 2 weeks before the first periodontal examination and, until the end of this study, all participants were uniformly instructed to brush their teeth using a soft toothbrush twice per day in accordance with the BASS Technique. SRP was performed immediately after the periodontal examinations at baseline and at 6 and 12 months. As a personalized strategy based on clinical characteristics, risk factors, and status of oral plaque, supportive periodontal therapy was maintained for at least 1 year, aimed to prevent periodontal reinfection and progression of periodontitis, avoid tooth loss, and timely detection and treatment of other oral disorders. For perimenopausal patients who required PG supplementation, dihydroxyprogesterone tablet (10 mg) was taken orally after the first periodontal examination. The PG intervention began on the 15^th^ day of menstruation every month and lasted for 6 months, twice per day, for 10 days per month.

### Periodontal examination

Periodontal examinations were performed at baseline and at 6 and 12 months. Periodontal parameters, including pocket probing depth (PPD), clinical attachment level (CAL), sulcus bleeding index (SBI), and bleeding on probing (BOP), were measured using UNC 15 periodontal probes (Hu-Friedy, Chicago, IL, USA) in each tooth at six sites (mesio-, mid-, disto-buccal, and mesio-, mid-, disto-lingual points). PPD represents the distance from the gingival margin to the bottom of the gingival pocket, and CAL represents the distance from the bottom of the gingival pocket to the cementoenamel junction. SBI is scored according to six grades described by Muhlemann and Son in 1971 (27). BOP represents bleeding rate of each tooth after probing. Periodontal parameters were blindly examined by two senior stomatologists.

### Measurement of inflammatory markers

Inflammatory markers were measured 1 week before the first periodontal examination and 1 week after the reexaminations (6 and 12 months). GCF samples were collected using paper strips from 6 target teeth (11, 16, 26, 31, 36, and 46) at the mesial, buccal, lingual, and distal sites. The levels of CRP, IL-6, and TNF-α in GCF were measured using commercially available ELISA kits (ThermoFisher Scientific, Waltham, MA, USA) in accordance with manufacturer’s instructions.

### Statistical analysis

PPD was used as the primary variable to calculate the sample size (24). To achieve 90% power with a 95% confidence interval (α = 0.05), the minimum sample size was 11. Statistical analyses were performed using SPSS version 21.0 (IBM Corporation, Armonk, NY, USA). Continuous data with normal distribution are expressed as mean ± standard deviation. Differences among multiple groups were analyzed using two-way analysis of variance and Tukey’s test for pairwise comparisons. Differences with P < 0.05 were considered to be statistically significant.

## Results

### Effects of PG on periodontal destruction in women with periodontitis who did or did not undergo SRP

Two key periodontal parameters—PPD and CAL—were used to evaluate periodontal destruction among different groups of women with periodontitis. As shown in **Table 1**, PPD was significantly lower in the SRP group than in the control group, and was also significantly lower in the SRP + PG group than in the control + PG group at 6 and 12 months (P < 0.001). In control patients who did not undergo SRP, PPD was significantly increased after a 6-month intervention with PG (P < 0.05). However, PPD partially decreased 6 months after withdrawal of PG and was not significantly different between the control and control + PG groups at 12 months. In patients who underwent SRP, PPD was slightly increased after 6 months of PG, although the difference was not statistically significant. At 12 months, PPD in the SRP + PG group decreased to a level similar to that of the SRP group. Consistent with PPD, CAL was significantly decreased by SRP with or without PG (P < 0.001). Additionally, intervention with PG slightly decreased CAL among patients, regardless of whether they underwent SRP, at 6 and 12 months; however, the differences were not statistically significant (**Table 2**).

**Table 1:** Pocket probing depth (PPD, mm) in women with periodontitis undergoing SRP and/or PG.

Times	Control (N = 29)	Control + PG (N = 31)	SRP (N = 35)	SRP + PG (N = 34)
0 month	3.590 ± 0.523	3.534 ± 0.512	3.571 ± 0.475	3.636 ± 0.536
6 months	3.613 ± 0.529	3.949 ± 0.603^*^	2.548 ± 0.388^***^	2.772 ± 0.425^###^
12 months	3.694 ± 0.479	3.716 ± 0.503	2.489 ± 0.364^***^	2.407 ± 0.402^###^

^*^P < 0.05, ^***^P < 0.001 vs. Control; ^###^P < 0.001 vs. Control + PG.

**Table 2:** Clinical attachment level (CAL, mm) in women with periodontitis undergoing SRP and/or PG.

Times	Control (N = 29)	Control + PG (N = 31)	SRP (N = 35)	SRP + PG (N = 34)
0 month	2.963 ± 0.433	3.013 ± 0.460	2.989 ± 0.522	3.007 ± 0.594
6 months	3.024 ± 0.485	2.762 ± 0.529	2.265 ± 0.475^***^	2.114 ± 0.451^###^
12 months	3.145 ± 0.595	2.905 ± 0.588	2.158 ± 0.429^***^	2.011 ± 0.452^###^

^***^P < 0.001 vs. Control; ^###^P < 0.001 vs. Control + PG.

### Effects of PG on periodontal inflammation among women with periodontitis who did or did not undergo SRP

SBI and BOP, two important indicators of periodontal inflammation, were evaluated. In the presence or absence of PG, both SBI and BOP were significantly reduced by SRP in women with periodontitis at 6 and 12 months (P < 0.001). Intervention with PG for 6 months significantly increased SBI and BOP in control patients who did not undergo SRP (P < 0.01). Six months after withdrawal of PG (at 12 months), SBI and BOP in the control + PG group decreased to levels similar to those of the control group. In contrast, PG treatment for 6 months significantly decreased BOP (P < 0.05) and slightly decreased SBI in patients who underwent SRP. No significant differences were observed in BOP and SBI between the SRP and SRP + PG groups at 12 months (**Tables 3**, **4**).

**Table 3:** Sulcus bleeding index (SBI) in periodontitis women undergoing SRP and/or PG.

Times	Control (N = 29)	Control + PG (N = 31)	SRP (N = 35)	SRP + PG (N = 34)
0 month	3.062 ± 0.554	3.042 ± 0.507	3.085 ± 0.534	3.069 ± 0.528
6 months	3.074 ± 0.502	3.468 ± 0.480^**^	1.572 ± 0.280^***^	1.351 ± 0.290^###^
12 months	3.122 ± 0.431	3.111 ± 0.560	1.461 ± 0.302^***^	1.402 ± 0.298^###^

**P < 0.01 vs. Control; ^***^P < 0.001 vs. Control; ^###^P < 0.001 vs. Control + PG.

**Table 4:** Bleeding on probing (BOP) in periodontitis women undergoing SRP and/or PG.

Times	Control (N = 29)	Control + PG (N = 31)	SRP (N = 35)	SRP + PG (N = 34)
0 month	59.847 ± 9.303	58.155 ± 9.020	59.154 ± 8.900	60.646 ± 8.664
6 months	59.954 ± 9.133	68.687 ± 8.548^***^	35.103 ± 5.635^***^	30.128 ± 5.224^###^^
12 months	60.634 ± 8.542	62.640 ± 7.927	32.003 ± 5.308^***^	31.149 ± 5.622^###^

^***^P < 0.001 vs. Control; ^###^P < 0.001 vs. Control + PG, ^^^P < 0.001 vs. SRP.

### Effects of PG on inflammatory indictors in women with periodontitis who did or did not undergo SRP

**Figure 2:**
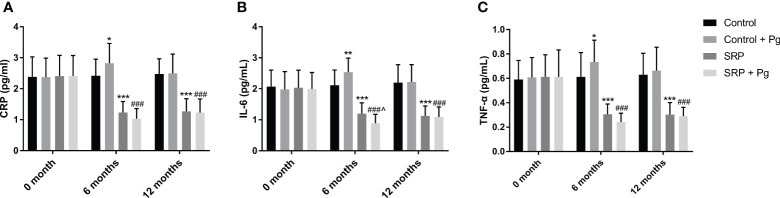
GCF levels of CRP **(A)**, IL-6 **(B)**, and TNF-α **(C)** in women with periodontitis undergoing SRP and/or PG (6 months). ^*^P < 0.05, ^**^P < 0.01, ^***^P < 0.001 vs. Control; ^###^P < 0.001 vs. Control + PG; ^^^P < 0.05 vs. SRP.

To further identify the differential effects of PG on periodontal inflammation in the presence or absence of SRP, the levels of three inflammatory indicators in GCF, including CRP, IL-6, and TNF-α, were measured. As shown in **Figures 2A–C**, GCF levels of CRP, IL-6, and TNF-α were significantly decreased by SRP with or without PG (P < 0.001). In control patients who did not undergo SRP, the levels of CRP, IL-6, and TNF-α were significantly increased after 6 months of PG treatment (P < 0.05). However, these indicators decreased 6 months after withdrawal of PG (at 12 months) and were not significantly different between the SRP and SRP + PG groups. In contrast with control patients, intervention with PG for 6 months significantly decreased IL-6 level (P < 0.05), and slightly decreased CRP and TNF-α levels in patients who underwent SRP. Six months after withdrawal of PG, none of these indicators were significantly different between the SRP and SRP + PG groups.

## Discussion

PG is a physiological partner of estrogen and is widely administered for a series of indications in females, such as assisted reproductive technology, anovulatory menstrual cycle, contraception during lactation, and postmenopausal endometrial hyperplasia (28). PG is commonly used to antagonize the adverse effects of estrogen (18, 19, 21). In this study, responses of the periodontal environment to PG were evaluated in perimenopausal women with periodontitis. Results demonstrated that a 6-month intervention with PG significantly increased PPD, SBI, and BOP. These results indicate an adverse role of PG in the periodontal environment, which is similar to previous findings in pregnant women. For example, Taani et al. found higher gingival index and PPD in pregnant women with high PG level than in non-pregnant women (29). Raga et al. revealed that decreasing PG after delivery is associated with a reduction in BOP, PPD, and CAL in pregnant women without periodontal treatment (30). Gil et al. reported decreased PG, BOP, and PPD levels in pregnant women after childbirth (31). Potential mechanisms underlying the adverse effects of PG on the periodontium are twofold. First, PG induces the hyperproliferation of periodontal ligament cells, contributing to gingival hyperplasia (32, 33). Second, PG exacerbates inflammation initiated by dental plaque (23). In addition, we found that CAL tended to decrease with PG by the intervention, although the difference was not significant. This phenomenon may be explained by the fact that PG inhibits alveolar bone loss in patients with periodontitis. PG is a “link” between the ovaries and bone in women. More specifically, PG level is positively associated with bone formation and negatively associated with bone resorption in perimenopausal women (34). Therefore, the protective effects of PG on the alveolar bone may offset its adverse effects on the periodontium. In addition, results of this study revealed that the effects of PG on the above periodontal parameters disappeared 6 months after withdrawal of PG. This phenomenon indicates the effects of PG on periodontitis are instantaneous.

SRP is a common nonsurgical strategy to clean subgingival biofilms and is indisputably effective for the treatment of periodontitis (35, 36). In this study, SRP significantly decreased PPD, CAL, SBI, and BOP, demonstrating its efficacy in improving periodontitis in perimenopausal women. Interestingly, we found that CAL and SBI were slightly decreased, and BOP was significantly decreased after 6 months of PG treatment in patients who underwent SRP. These results indicate that PG plays an anti-inflammatory role in the periodontal environment after SRP. These findings are consistent with those of previous studies investigating many other disorders. For example, PG inhibits inflammation, neovascularization, and neurogenesis during endometriosis (37). PG inhibits airway inflammation by synergistically interacting with glucocorticoids (38). PG suppresses inflammation and apoptosis to protect the brain from hypoxic-ischemic damage (39). Notably, the anti-inflammatory effect of PG in the presence of SRP was contrary to its pro-inflammatory effect in the absence of SRP. This difference may be attributed to the status of dental plaque. Dental plaque is a diverse microbial community present on the tooth surface, and its accumulation is a leading risk factor for the onset of periodontitis (40). Wu et al. reported that PG exacerbates periodontal inflammation initiated by dental plaque (23). Therefore, when dental plaque is cleared by SRP, PG may inhibit inflammation. In addition, after SRP treatment, we found a slight elevation in PPD in patients treated with PG, although the difference was not significant. This result is similar to findings in patients who did not undergo SRP and indicates that the promoting effect of PG on gingival hyperplasia persists after SRP.

In the mouth, various immune regulators are involved in the defense response against pathogenic bacteria, including pro-inflammatory cytokines, matrix metalloproteinases, and reactive oxygen species, all of which contribute to periodontal damage (41). IL-6 and TNF-α are common inflammatory cytokines that are elevated in patients with periodontitis (42). Our previous study found that 3 months of PG treatment elevates GCF levels of IL-6 and TNF-α in women with periodontitis (43). Accordingly, this study also revealed that IL-6 and TNF-α levels were increased by 6 months of treatment with PG in patients who did not undergo SRP. As the mainstay of periodontal therapy, SRP is associated with reduced GCF levels of inflammatory cytokines (44). In this study, SRP significantly decreased the GCF levels of IL-6 and TNF-α, which supports the anti-inflammatory effect of SRP in periodontitis. Of note, a significant decrease in IL-6 level and a slight decrease in TNF-α level were induced by PG in patients who underwent SRP. These results indicate that PG exerts an anti-inflammatory effect in the presence of SRP, which is different from its pro-inflammatory effect in the absence of SRP. These findings also support changes in SBI and BOP in patients with periodontitis, demonstrating that PG plays a “double-edged” role in periodontal inflammation by regulating inflammatory cytokines. In addition, CRP is an acute-phase protein produced in response to infection or damage and an inflammatory marker (45). Elevated CRP level is closely associated with the onset of periodontitis (46). Evidence has demonstrated that SRP is effective in reducing CRP level (47), which is consistent with results obtained in this study. In addition, Gil et al. reported that CRP level increases during pregnancy with high PG level, which is positively correlated with BOP (31). Consistent with BOP, CRP level also significantly increased 6 months after PG in patients who did not undergo SRP. In contrast, CRP was slightly decreased by PG in patients who underwent SRP. Therefore, CRP also exhibits different responses to PG in perimenopausal women with periodontitis in the presence or absence of SRP.

## Conclusion

SRP relieved periodontitis in perimenopausal women. Intervention with PG instantaneously enhanced periodontal inflammation in the absence of SRP but inhibited it in the presence of SRP. Active SRP is recommended for perimenopausal women with periodontitis, and PG can further enhance the inhibitory effect of SRP on inflammation. However, this study is limited by an insufficient sample size. As such, the mechanisms of action of PG in periodontitis related to dental plaque need to be further explored.

## Data availability statement

The original contributions presented in the study are included in the article/supplementary material. Further inquiries can be directed to the corresponding authors.

## Ethics statement

The studies involving human participants were reviewed and approved by Ethics Committee of Shengli Oilfield Central Hospital (Q/ZXYY-ZY-YWBLL-201819). The patients/participants provided their written informed consent to participate in this study.

## Author contributions

Conception and design: SY, YM, FN, and JQ; Data collection: SY, YM, LC, FN, and JQ; Data analysis and interpretation: SY, YM, and JL; Drafting the manuscript: SY and YM; Revising the manuscript critically for important intellectual content: FN and JQ; Funding acquisition: YM; All authors contributed to the article and approved the submitted version.
